# Vitamin B12 Deficiency Without Anemia Presenting With Delayed Orthostatic Hypotension in an Adolescent: A Case Report

**DOI:** 10.1002/ccr3.73338

**Published:** 2026-08-12

**Authors:** Kenshin Tanaka, Shun Yamashita, Kota Minami, Yumi Harano, Masaki Tago

**Affiliations:** ^1^ Department of Urology, Faculty of Medicine Saga University Saga Japan; ^2^ Department of General Medicine Saga University Hospital Saga Japan; ^3^ Education and Research Center for Community Medicine, Faculty of Medicine Saga University Saga Japan; ^4^ Department of Medicine Mount Sinai Morningside and West New York USA; ^5^ Department of General Internal Medicine Local Incorporated Administrative Agency Saga‐Ken Medical Centre Koseikan Saga Japan

**Keywords:** active standing test, adolescent female, anti‐intrinsic factor antibodies, orthostatic hypotension, vitamin B12 deficiency

## Abstract

The orthostatic hypotension caused by vitamin B12 deficiency is extremely rare in young individuals. The serum vitamin B12 and anti‐intrinsic factor antibody levels of young patients with orthostatic hypotension should be evaluated regardless of the presence of anemia, and vitamin B12 administration should be promptly initiated.

## 1 Introduction

Vitamin B12, which is involved in neural conduction, is essential for DNA synthesis in hematopoietic cells and myelination [1]. The incidence of vitamin B12 deficiency is approximately 3%–6% among the general population; however, this rate varies by age group [2]. Common causes include inadequate intake attributable to restrictive eating, malabsorption syndromes, and medication‐induced deficiency attributable to prolonged use of acid‐suppressing agents [2, 3]. Vitamin B12 deficiency presents with extremely diverse symptoms, including generalized fatigue, dyspnea, distal paresthesia, gait disturbance, glossitis, dysgeusia, and anorexia, and leads to impaired hematopoiesis and disordered methylation of myelin‐forming cells, which result in megaloblastic anemia, peripheral neuropathy, and demyelination of the posterior columns of the spinal cord [4]. Some patients present with orthostatic hypotension (OH), which is associated with autonomic dysfunction caused by inadequate sympathetic vasoconstriction and reduced heart rate variability (HRV) when standing [5]. The diagnosis of OH can be challenging when patients are at low risk for vitamin B12 deficiency, present with isolated neurological symptoms without anemia, or present without macrocytic anemia [6, 7].

Vitamin B12 deficiency should be considered as a potential cause of adolescent OH, which has broad differential diagnoses. Although vitamin B12 deficiency caused by intrinsic factor antibodies is often observed in older adults, it is extremely rare in young patients. We report the case of an adolescent female patient with orthostatic hypotension caused by vitamin B12 deficiency attributable to intrinsic factor antibodies, highlighting the importance of checking vitamin B12 levels in young OH patients even without anemia.

## 2 Case History/Examination

Our patient was a 17‐year‐old girl who had no underlying diseases, no evidence of reduced circulating plasma volume, no history of restrictive eating, and no gastrointestinal symptoms such as diarrhea. She did not use acid suppressants, medications, or supplements, and her menses were regular. Two years before presentation, she experienced visual dimming immediately upon standing after waking followed by unconsciousness for 5 min. Although her symptoms improved, she experienced two similar syncopal episodes at age 17 years without accompanying symptoms such as vomiting or sweating, and promptly presented to a cardiology clinic. Ambulatory electrocardiographic monitoring showed the absence of arrhythmia; however, transthoracic echocardiography demonstrated mild mitral and tricuspid regurgitation, normal ejection fraction, and no regional wall motion abnormalities. One month after the initial assessment, she was referred to our department of general internal medicine for further evaluation of syncope.

On arrival, the patient was alert. Her vital signs were as follows: body temperature, 36.6°C; pulse rate, 76 beats/min; blood pressure in the seated position, 111/71 mmHg; respiratory rate, 18 breaths/min; and oxygen saturation, 99% on room air. Her height and weight were 159 cm and 51.7 kg, and her body mass index was 20.2. A physical examination revealed no conjunctival pallor, thyroid enlargement, or oral dryness. Cardiac and pulmonary sounds were unremarkable. The abdomen was flat and soft; tenderness and hepatosplenomegaly were not observed. Visual field defects, paralysis, impaired pain, impaired temperature sensation, and gait disturbances were absent.

## 3 Differential Diagnosis, Investigations, and Treatment

**FIGURE 1:**
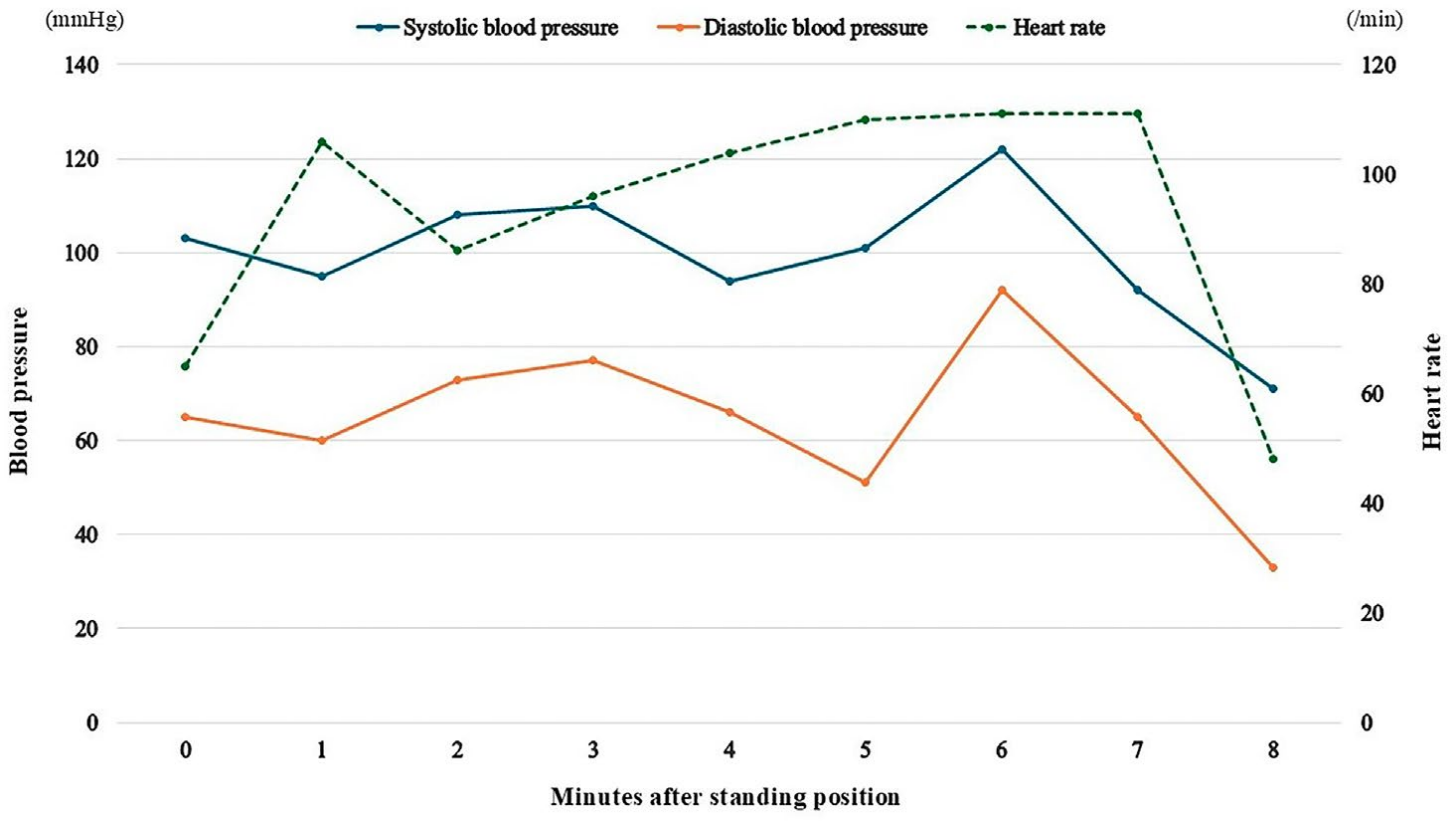
Orthostatic tolerance test results before vitamin B12 administration. Systolic blood pressure decreased by 32 mmHg (from 103 to 71 mmHg) at 8 min after standing.

Laboratory tests at the time of admission indicated the following: white blood cell count, 7900/μL; neutrophils, 67%; hemoglobin, 12.8 g/dL; mean corpuscular volume, 91 fL; mean corpuscular hemoglobin concentration, 33.8 g/dL; platelet count, 24.5 × 10^4^/μL; C‐reactive protein, 0.01 mg/dL; blood urea nitrogen, 9.5 mg/dL; creatinine, 0.62 mg/dL; sodium, 140 mEq/L; potassium, 3.5 mEq/L; and chloride, 108 mEq/L (Table 1). A peripheral blood smear did not reveal any megaloblastic changes. Although thyroid function and the serum cortisol level were normal, the serum adrenocorticotropic hormone level was mildly decreased (5.8 pg/mL). A rapid adrenocorticotropic hormone stimulation test and 24‐h urinary free cortisol test yielded normal results. Chest radiography revealed the absence of cardiomegaly and pleural effusion. Electrocardiography revealed sinus arrhythmia with a heart rate of 54 beats/min. Cranial magnetic resonance imaging (MRI) without contrast enhancement revealed an incidental Rathke's cleft cyst, which was considered unrelated to the patient's orthostatic hypotension. The active standing test was stopped because the patient's systolic blood pressure decreased by 32 to 71/33 mmHg after 8 min (Figure 1).

**TABLE 1:** Laboratory findings on admission.

Complete blood count		Biochemistry		Biochemistry	
WBC	7.9 × 10^3^/μL	AST	14 IU/L	Prolactin	13.1 ng/mL
Neutrophil	67%	ALT	10 IU/L	GH	0.61 ng/dL
Red blood cell	415 × 10^4^/μL	ALP	55 IU/L	TSH	0.38 mIU/L
Hemoglobin	12.8 g/dL	LDH	162 IU/L	FT4	1.00 ng/mL
MCV	91.3 fL	CK	71 IU/L	ACTH	5.8 pg/mL
MCHC	33.8 g/dL	CRP	0.01 mg/dL	Cortisol	5.3 μg/dL
Platelet	24.5 × 10^4^/μL	Sodium	140 mmol/L	Copper	69 μg/dL
Biochemistry		Potassium	3.5 mmol/L		4.6 Zinc
Total protein	6.3 g/dL	Chloride	108 mmol/L	Vitamin B12	105 pg/mL
Albumin	4.0 g/dL	IGF‐1	358 μg/dL	Folic acid	4.6 ng/mL
BUN	9.5 mg/dL	LH	4.94 mIU/mL	Anti‐gastric parietal cell antibody	(−)
Creatinine	0.62 mg/dL	FSH	4.40 IU/mL	Anti‐intrinsic factor antibody	(+)

Abbreviations: ACTH, adrenocorticotropic hormone; ALP, alkaline phosphatase; ALT, alanine aminotransferase; AST, aspartate aminotransferase; BUN, blood urea nitrogen; CK, creatine kinase; CRP, C‐reactive protein; FSH, follicle‐stimulating hormone; FT4, free thyroxine 4; GH, growth hormone; IGF‐1, insulin‐like growth factor 1; LDH, lactate dehydrogenase; LH, luteinizing hormone; MCHC, mean corpuscular hemoglobin concentration; MCV, mean corpuscular volume; TSH, thyroid‐stimulating hormone; WBC, white blood cell.

This finding resulted in a diagnosis of delayed orthostatic hypotension, in which a blood pressure drop meeting orthostatic criteria is observed more than 3 min after standing [8]. Given the marked blood pressure reduction and the patient's history of recurrent syncope, additional autonomic function testing such as head‐up tilt testing was not performed because it could have induced severe hypotension and syncope. Additionally, the markedly decreased serum vitamin B12 level (105 pg/mL) and presence of intrinsic factor antibodies led to the diagnosis of delayed OH caused by vitamin B12 deficiency associated with intrinsic factor antibodies.

The patient received an intramuscular injection of vitamin B12 500 μg daily for 3 consecutive days immediately after diagnosis and was discharged on hospital Day 4.

## 4 Outcome and Follow Up

Five days after discharge, the serum vitamin B12 level was markedly elevated (10,200 pg/mL). Thereafter, intramuscular administration of the same dose of vitamin B12 was continued once per month. Four months later, a repeat active standing test showed a 10‐mmHg decrease in systolic blood pressure (from 108 to 98 mmHg). Although syncope no longer occurred, dizziness and visual dimming persisted.

## 5 Discussion

OH is classified as primary or secondary. The representative causes of primary OH include Parkinson disease, multiple system atrophy, and Lewy body dementia [8]. The representative causes of secondary OH include diabetic or alcoholic neuropathy, reduced circulating plasma volume, cardiac diseases, endocrine disorders, and adverse effects of medications [8]. Vitamin B12 deficiency can cause sensory impairment and secondary autonomic dysfunction by affecting small myelinated Aδ fibers and unmyelinated C fibers [9]. The prevalence of vitamin B12 deficiency is approximately 2.6%–7.5% among the general population and 1.3%–2.6% among young individuals [10]. Its manifestations are diverse and include psychiatric disorders, dysautonomia, and orthostatic intolerance such as OH [11]. The main causes of vitamin B12 deficiency are inadequate nutrition intake, malabsorption syndrome, and the effects of medications [12, 13]. Although the presence of intrinsic factor antibodies is also a contributing cause, the presence is often observed in elderly patients and is extremely rare among young patients [8, 14]. When a young patient with OH presents without any underlying disease, vitamin B12 deficiency should be suspected after major secondary causes have been excluded. Additionally, when a common cause of vitamin B12 deficiency is not identified, testing for intrinsic factor antibodies is warranted.

A key feature of this case was the absence of macrocytic anemia and neurological abnormalities other than OH despite vitamin B12 deficiency. Vitamin B12 deficiency commonly causes macrocytic anemia; in some cases, the mean corpuscular volume can exceed 120 fL [15]. However, approximately 10% of patients with normocytic anemia have vitamin B12 deficiency [15]. Therefore, the absence of macrocytic anemia alone cannot exclude vitamin B12 deficiency. Furthermore, among patients with vitamin B12 deficiency, it is not uncommon to present with only neurological symptoms in the absence of anemia [6]. More than 25% of patients with intrinsic factor antibodies develop only neurological manifestations in the absence of anemia or megaloblastic changes [15, 16]. A case of OH without anemia or other neurological abnormalities has been reported previously [17]. Screening for vitamin B12 deficiency based on the assumption of macrocytic anemia or neurological signs other than OH may lead to a delayed or missed diagnosis.

Vitamin B12 replacement therapy is the primary treatment for OH caused by vitamin B12 deficiency [18]. Orthostatic syncope caused by vitamin B12 deficiency in elderly patients can be improved with replacement therapy [18]. A decreased coefficient of variation of R‐R intervals on electrocardiography suggesting reduced parasympathetic activity in patients with vitamin B12 deficiency can also be improved with vitamin B12 replacement [19]. Thus, early vitamin B12 replacement therapy is expected to improve the symptoms and prognosis of vitamin B12 deficiency. However, delaying treatment initiation can result in limited symptom improvement and persistent residual deficits [20]. Complete recovery can be difficult if several months or more have elapsed between symptom onset and treatment initiation [21]. Involvement of the central nervous system, including the spinal cord or brain, can be irreversible [21]. In our case, more than 2 years had passed between symptom onset and treatment initiation. Although syncope has not recurred, dizziness and visual dimming may persist. Therefore, prompt diagnosis and treatment initiation are important to improving patient outcomes.

In this case, differentiating OH from vasovagal syncope (VVS) was the most challenging issue. VVS is the most common cause of syncope, and approximately 40% of adults experience at least one episode during their lifetime [22]. VVS is characterized by an abrupt fall in blood pressure and heart rate triggered by prolonged standing, pain stimuli, or emotional stress, and is typically accompanied by symptoms such as dizziness, blurred vision, vomiting, sweating, palpitations, and a feeling of weakness during syncope [23]. In contrast, OH leads to a fall in blood pressure immediately or more than 3 min after standing, resulting in syncope, and its occurrence in young adults is rare [24, 25]. Furthermore, OH may be followed by VVS, and in such cases, the concomitant bradycardia during hypotension makes distinguishing OH from VVS particularly difficult [25]. In the present case, all previous syncopal episodes occurred immediately after standing without accompanying symptoms and vitamin B12 supplementation resolved both the orthostatic blood pressure drop and the syncope. Based on these findings, we diagnosed OH. However, because bradycardia was observed during the blood pressure fall, complete exclusion of VVS remains challenging.

This case involved some limitations. Upper gastrointestinal endoscopy was not performed, and underlying causes of intrinsic factor antibodies were not fully differentiated. Additionally, a central nervous system abnormality, such as subacute combined degeneration of the spinal cord, involved in vitamin B12 deficiency could not be ruled out in this case because spinal MRI was not performed. If MRI had demonstrated these findings, then a more reliable prognostic assessment may have been possible. Finally, further autonomic function testing beyond the active standing test, such as head‐up tilt testing, was not performed in the present case. Such evaluation might have strengthened the diagnostic assessment.

## 6 Conclusion

Vitamin B12 deficiency attributable to intrinsic factor antibodies was considered the most likely cause of delayed OH in our adolescent patient. When a young patient with OH presents without underlying disease, a serum vitamin B12 test should be performed, regardless of the presence of macrocytic anemia, and early treatment should be initiated to improve outcomes.

## Author Contributions

**Kenshin Tanaka:** conceptualization, resources, writing – original draft. **Shun Yamashita:** conceptualization, supervision, writing – original draft. **Yumi Harano:** conceptualization, resources. **Kota Minami:** conceptualization, writing – original draft. **Masaki Tago:** supervision, writing – review and editing.

## Funding

The authors have nothing to report.

## Ethics Statement

This manuscript conforms to the provisions of the Declaration of Helsinki in 1995 (as revised in Brazil 2013).

## Consent

Written informed consent was obtained from the patient to publish this report.

## Conflicts of Interest

The authors declare no conflicts of interest.

## Data Availability

The data that support the findings of this study are available from the corresponding author upon reasonable request.

## References

[1] C. A. Calderón‐Ospina and M. O. Nava‐Mesa , “B Vitamins in the Nervous System: Current Knowledge of the Biochemical Modes of Action and Synergies of Thiamine, Pyridoxine, and Cobalamin,” CNS Neuroscience & Therapeutics 26 (2020): 5–13. 31490017 10.1111/cns.13207 PMC6930825

[2] M. J. Shipton and J. Thachil , “Vitamin B12 Deficiency—A 21st Century Perspective,” Clinical Medicine (London, England) 15 (2015): 145–150. 25824066 10.7861/clinmedicine.15-2-145 PMC4953733

[3] H. Mumtaz , B. Ghafoor , H. Saghir , et al., “Association of Vitamin B12 Deficiency With Long‐Term PPIs Use: A Cohort Study,” Annals of Medicine and Surgery 82 (2022): 104762. 36268318 10.1016/j.amsu.2022.104762 PMC9577826

[4] J. Singh , A. Dinkar , P. Gupta , and V. Atam , “Vitamin B12 Deficiency in Northern India Tertiary Care: Prevalence, Risk Factors and Clinical Characteristics,” Journal of Family Medicine and Primary Care 11 (2022): 2381–2388. 10.4103/jfmpc.jfmpc_650_21 PMC9480660 36119310

[5] M. Beitzke , P. Pfister , J. Fortin , and F. Skrabal , “Autonomic Dysfunction and Hemodynamics in Vitamin B12 Deficiency,” Autonomic Neuroscience 97 (2002): 45–54. 12036186 10.1016/s1566-0702(01)00393-9

[6] A. Mesgarankarimi , M. Rezapour , and N. Tabrizi , “A Long‐Standing Undiagnosed Case of Vitamin B12 Deficiency: A Case Report,” Journal of Medical Case Reports 19 (2025): 151. 40176197 10.1186/s13256-025-05149-7 PMC11963364

[7] D. M. P. U. K. Ralapanawa , K. P. Jayawickreme , E. M. M. Ekanayake , and W. A. T. A. Jayalath , “B12 Deficiency With Neurological Manifestations in the Absence of Anaemia,” BMC Research Notes 8 (2015): 458. 26385097 10.1186/s13104-015-1437-9 PMC4575440

[8] F. Ricci , R. De Caterina , and A. Fedorowski , “Orthostatic Hypotension: Epidemiology, Prognosis, and Treatment,” Journal of the American College of Cardiology 66 (2015): 848–860. 26271068 10.1016/j.jacc.2015.06.1084

[9] D. Agrican , N. Bal , T. Gesoglu‐Demir , A. Gocmen , and O. Ethemoglu , “The Electrophysiological Evaluation of Small Fiber Neuropathy in Symptomatic Patients With Vitamin B12 Deficiency Before and After Treatment,” Neurological Sciences and Neurophysiology 41 (2024): 182–189.

[10] J. K. Bird , R. A. Murphy , E. D. Ciappio , and M. I. McBurney , “Risk of Deficiency in Multiple Concurrent Micronutrients in Children and Adults in the United States,” Nutrients 9 (2017): 655. 28672791 10.3390/nu9070655 PMC5537775

[11] H. M. Serin and E. A. Arslan , “Neurological Symptoms of Vitamin B12 Deficiency: Analysis of Pediatric Patients,” Acta Clinica Croatica 58 (2019): 295–302. 31819326 10.20471/acc.2019.58.02.13 PMC6884369

[12] A. R. Mathew , G. Di Matteo , P. La Rosa , et al., “Vitamin B12 Deficiency and the Nervous System: Beyond Metabolic Decompensation‐Comparing Biological Models and Gaining New Insights Into Molecular and Cellular Mechanisms,” International Journal of Molecular Sciences 25 (2024): 590. 38203763 10.3390/ijms25010590 PMC10778862

[13] W. Herrmann and R. Obeid , “Causes and Early Diagnosis of Vitamin B12 Deficiency,” Deutsches Ärzteblatt International 105 (2008): 680–685. 19623286 10.3238/arztebl.2008.0680 PMC2696961

[14] Saif‐ur‐Rehman , L. Zafar , T. Imran , A. Ghafoor , A. A. Durrani , and T. A. Ahmed , “Frequency of Intrinsic Factor Antibody in Megaloblastic Anaemia,” Journal of the College of Physicians and Surgeons–Pakistan 24 (2014): 157–159. 24613108

[15] S. P. Stabler , “Vitamin B_12_ Deficiency,” New England Journal of Medicine 368 (2013): 149–160. 23697526 10.1056/NEJMc1304350

[16] J. Lindenbaum , E. B. Healton , D. G. Savage , et al., “Neuropsychiatric Disorders Caused by Cobalamin Deficiency in the Absence of Anemia or Macrocytosis,” New England Journal of Medicine 318 (1988): 1720–1728. 3374544 10.1056/NEJM198806303182604

[17] Z. Yousaf , A. Razok , A. N. Elzouki , and T. Sabobeh , “Hypotension: An Unusual Presentation of Vitamin B_12_ Deficiency, With Complete Recovery Following Cyanocobalamin Therapy,” BMJ Case Reports 12 (2019): e232677. 10.1136/bcr-2019-232677 PMC6904166 31811099

[18] L. Ganjehei , A. Massumi , M. Razavi , and J. M. Wilson , “Orthostatic Hypotension as a Manifestation of Vitamin B12 Deficiency,” Texas Heart Institute Journal 39 (2012): 722–723. 23109778 PMC3461697

[19] K. Aytemir , S. Aksöyek , Y. Büyükasik , et al., “Assessment of Autonomic Nervous System Functions in Patients With Vitamin B12 Deficiency by Power Spectral Analysis of Heart Rate Variability,” Pacing and Clinical Electrophysiology 23 (2000): 975–978. 10879381 10.1111/j.1540-8159.2000.tb00883.x

[20] K. K. Jain , H. S. Malhotra , R. K. Garg , P. K. Gupta , B. Roy , and R. K. Gupta , “Prevalence of MR Imaging Abnormalities in Vitamin B12 Deficiency Patients Presenting With Clinical Features of Subacute Combined Degeneration of the Spinal Cord,” Journal of the Neurological Sciences 342 (2014): 162–166. 24857760 10.1016/j.jns.2014.05.020

[21] O. M. Vasconcelos , E. H. Poehm , R. J. McCarter , W. W. Campbell , and Z. M. N. Quezado , “Potential Outcome Factors in Subacute Combined Degeneration,” Journal of General Internal Medicine 21 (2006): 1063–1068. 16970556 10.1111/j.1525-1497.2006.00525.x PMC1831618

[22] P. Kalhor , P. Ghahari , N. Asgari , A. Jalali , and S. Sadeghian , “A Tick of the Clock: Finding the Sweet Spot in Tilt Table Test. The Effectiveness of Short‐Duration Head‐Up Tilt Test as a Diagnostic Tool in Suspected Vasovagal Patients: A Retrospective Observational Study in a Tertiary Syncope Unit,” Journal of Arrhythmia 41 (2025): e70190. 41019583 10.1002/joa3.70190 PMC12463703

[23] B. Malave and B. Vrooman , “Vasovagal Reactions During Interventional Pain Management Procedures‐A Review of Pathophysiology, Incidence, Risk Factors, Prevention, and Management,” Medicina (Basel) 10 (2022): 39. 10.3390/medsci10030039 PMC9332485 35893121

[24] D. J. L. van Twist , M. P. M. Harms , V. K. van Wijnen , et al., “Diagnostic Criteria for Initial Orthostatic Hypotension: A Narrative Review,” Clinical Autonomic Research 31 (2021): 685–698. 34677720 10.1007/s10286-021-00833-2

[25] A. Fedorowski , V. K. van Wijnen , and W. Wieling , “Delayed Orthostatic Hypotension and Vasovagal Syncope: A Diagnostic Dilemma,” Clinical Autonomic Research 27 (2017): 289–291. 28550504 10.1007/s10286-017-0424-8

